# Mapping behavioural, cognitive and affective transdiagnostic dimensions in frontotemporal dementia

**DOI:** 10.1093/braincomms/fcac344

**Published:** 2023-01-05

**Authors:** Siddharth Ramanan, Hashim El-Omar, Daniel Roquet, Rebekah M Ahmed, John R Hodges, Olivier Piguet, Matthew A Lambon Ralph, Muireann Irish

**Affiliations:** Medical Research Council Cognition and Brain Sciences Unit, The University of Cambridge, Cambridge CB3 1AU, UK; Brain and Mind Centre, The University of Sydney, Sydney, NSW 2050, Australia; School of Psychology, The University of Sydney, Sydney, NSW 2050, Australia; Brain and Mind Centre, The University of Sydney, Sydney, NSW 2050, Australia; Brain and Mind Centre, The University of Sydney, Sydney, NSW 2050, Australia; School of Psychology, The University of Sydney, Sydney, NSW 2050, Australia; Brain and Mind Centre, The University of Sydney, Sydney, NSW 2050, Australia; Memory and Cognition Clinic, Department of Clinical Neurosciences, Royal Prince Alfred Hospital, Sydney, NSW 2050, Australia; Brain and Mind Centre, The University of Sydney, Sydney, NSW 2050, Australia; School of Psychology, The University of Sydney, Sydney, NSW 2050, Australia; School of Medical Sciences, The University of Sydney, Sydney, NSW 2050, Australia; Brain and Mind Centre, The University of Sydney, Sydney, NSW 2050, Australia; School of Psychology, The University of Sydney, Sydney, NSW 2050, Australia; Medical Research Council Cognition and Brain Sciences Unit, The University of Cambridge, Cambridge CB3 1AU, UK; Brain and Mind Centre, The University of Sydney, Sydney, NSW 2050, Australia; School of Psychology, The University of Sydney, Sydney, NSW 2050, Australia

**Keywords:** semantic variant primary progressive aphasia, principal component analysis, dementia, transdiagnostic, heterogeneity

## Abstract

Two common clinical variants of frontotemporal dementia are the behavioural variant frontotemporal dementia, presenting with behavioural and personality changes attributable to prefrontal atrophy, and semantic dementia, displaying early semantic dysfunction primarily due to anterior temporal degeneration. Despite representing independent diagnostic entities, mounting evidence indicates overlapping cognitive–behavioural profiles in these syndromes, particularly with disease progression. Why such overlap occurs remains unclear. Understanding the nature of this overlap, however, is essential to improve early diagnosis, characterization and management of those affected. Here, we explored common cognitive–behavioural and neural mechanisms contributing to heterogeneous frontotemporal dementia presentations, irrespective of clinical diagnosis. This transdiagnostic approach allowed us to ascertain whether symptoms not currently considered core to these two syndromes are present in a significant proportion of cases and to explore the neural basis of clinical heterogeneity. Sixty-two frontotemporal dementia patients (31 behavioural variant frontotemporal dementia and 31 semantic dementia) underwent comprehensive neuropsychological, behavioural and structural neuroimaging assessments. Orthogonally rotated principal component analysis of neuropsychological and behavioural data uncovered eight statistically independent factors explaining the majority of cognitive–behavioural performance variation in behavioural variant frontotemporal dementia and semantic dementia. These factors included Behavioural changes, Semantic dysfunction, General Cognition, Executive function, Initiation, Disinhibition, Visuospatial function and Affective changes. Marked individual-level overlap between behavioural variant frontotemporal dementia and semantic dementia was evident on the Behavioural changes, General Cognition, Initiation, Disinhibition and Affective changes factors. Compared to behavioural variant frontotemporal dementia, semantic dementia patients displayed disproportionate impairment on the Semantic dysfunction factor, whereas greater impairment on Executive and Visuospatial function factors was noted in behavioural variant frontotemporal dementia. Both patient groups showed comparable magnitude of atrophy to frontal regions, whereas severe temporal lobe atrophy was characteristic of semantic dementia. Whole-brain voxel-based morphometry correlations with emergent factors revealed associations between fronto-insular and striatal grey matter changes with Behavioural, Executive and Initiation factor performance, bilateral temporal atrophy with Semantic dysfunction factor scores, parietal-subcortical regions with General Cognitive performance and ventral temporal atrophy associated with Visuospatial factor scores. Together, these findings indicate that cognitive–behavioural overlap (i) occurs systematically in frontotemporal dementia; (ii) varies in a graded manner between individuals and (iii) is associated with degeneration of different neural systems. Our findings suggest that phenotypic heterogeneity in frontotemporal dementia syndromes can be captured along continuous, multidimensional spectra of cognitive–behavioural changes. This has implications for the diagnosis of both syndromes amidst overlapping features as well as the design of symptomatic treatments applicable to multiple syndromes.

## Teaser

Behavioural variant frontotemporal dementia and semantic dementia display co-occurring changes across multiple domains of behaviour, cognitive and affective functions that reflect graded, inter-individual variations along a multidimensional space of cognitive–behavioural functions and are underpinned by degeneration of overlapping regions within distributed neural networks.

## Graphical Abstract

**Graphical abstract:**
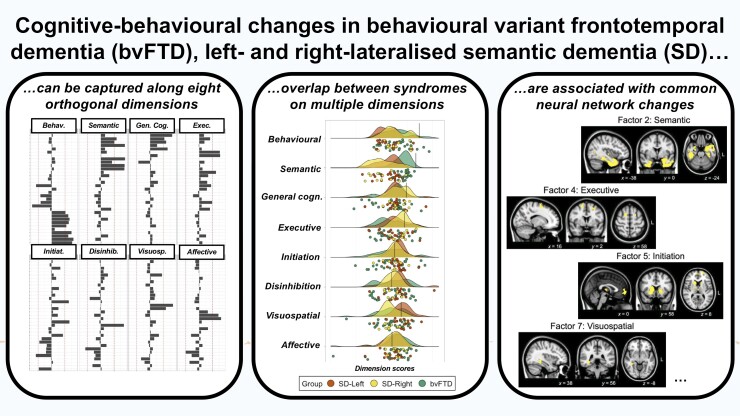

## Introduction

Frontotemporal dementia (FTD) refers to a group of rare, young-onset, progressive neurodegenerative brain disorders, primarily affecting the frontal and/or temporal lobes.^1^ Three clinical variants have been defined: a behavioural variant FTD (bvFTD), a semantic variant (also known as semantic variant primary progressive aphasia; here, referred to as semantic dementia or SD), both of which form the focus of the current study, and a non-fluent aphasic variant. Briefly, bvFTD presents with marked changes in behaviour, personality, executive and social cognition in the context of marked prefrontal and insular degeneration.^2,3^ In contrast, SD is characterized by profound anomia and comprehension difficulties reflecting trans-modal, trans-category loss of semantic knowledge arising from degeneration of bilateral ventral and anterior temporal lobes (ATL).^4–6^ While most SD patients present with left-predominant ATL degeneration (SD-Left), a right-lateralized pattern (SD-Right) has also been described, whereby atrophy initially targets the right hemisphere before producing a bilateral profile of ATL degeneration. These patients typically present with face-processing disturbances, socio-emotional dysfunction, behavioural changes and loss of insight, in addition to semantic impairments.^7–9^

Clinically, bvFTD and SD are considered as separate diagnostic entities; however, mounting evidence suggests the need to rethink traditional phenotypic boundaries between these syndromes,^10^ especially in terms of cognitive and behavioural performance.^9,11–13^ For example, semantic and language changes also occur in bvFTD^14–16^ and are detectable in early disease stages.^11^ Likewise, SD patients (especially SD-Right) can present with considerable socio-emotional and behavioural disturbances^8,17–21^ often of the same magnitude as bvFTD, which intensify with disease progression. This particular pattern of overlap can pose considerable challenges in the diagnostic distinction of SD-Right from bvFTD.^8,18^ Estimating the frequency of heterogeneous symptoms from clinical reports, Coyle-Gilchrist *et al*.^22^ found ∼74% of their referred bvFTD patients (*N* = 31/42) to show non-specific language difficulties, whereas ∼96% of their SD cohort (*N* = 22/23) displayed behavioural changes. Emerging evidence also suggests considerable overlap between bvFTD and SD on executive, attentional and visuospatial performance.^18^ Currently, it remains unclear why distinct clinical syndromes display overlapping symptoms. Two proposals relevant to FTD are that (i) emergent heterogeneity relates closely to advancing disease severity and/or duration and (ii) individuals showing a combination of bvFTD and SD symptoms (with additional prosopagnosia, driven in part by conceptual loss of people information, and visuospatial dysfunction) represent a clinically distinct entity such as the ‘right-temporal variant of FTD’.^12,23–25^ Both proposals, however, cannot account for ‘atypical’ symptoms in individuals early in the disease trajectory, in patients whose clinical profiles otherwise squarely meet diagnostic criteria for prototypical bvFTD or SD, or for the many patients presenting with ‘mixed’ symptomatology. As these overlaps are not reflected in international diagnostic criteria for bvFTD and SD, quantifying the nature and extent of such variation is important for improved diagnosis and characterization of both syndromes.

Cognitive–behavioural variations in bvFTD and SD have typically been studied using group comparisons of prevalence/severity of specific deficits. This approach is limited in its capacity to capture individual-level variability, subtle overlaps and fuzzy boundaries between both syndromes. Instead, transdiagnostic approaches that capture systematic, non-random variations in cognitive–behavioural symptoms across individuals, irrespective of categorical diagnostic labels, offer greater promise in this regard. By cutting across categorical boundaries, this approach aids examination of prevalence and magnitude of cognitive–behavioural features and their likely associations with the presenting phenotype.^26^ By moving from a diagnosis-centred to a symptom-centred approach, we can accommodate features that occur systematically in some patients (e.g. ‘pure’ presentations of bvFTD and SD) as well as symptoms that are ‘diagnostically atypical’ (e.g. occurring in those with ‘mixed profiles’).

Transdiagnostic approaches have been applied consistently in neuropsychiatry and post-stroke aphasia fields to model neurocognitive mechanisms explaining cognitive, behavioural and functional breakdowns, irrespective of syndrome-specificity.^27–30^ Recent studies employing these methods in dementia syndromes demonstrate considerable success in explaining symptomatic heterogeneity in terms of coherent variations along orthogonal dimensions of clinical and cognitive changes.^9,13,31–35^ Across syndromes, such phenotypic variations further closely relate to unique patterns of neural network degeneration,^9,13,32,35^ metabolic brain changes^36^ and categorically distinct neuropathological drivers of underlying disease.^37^ Applying transdiagnostic approaches in bvFTD and SD, therefore, holds immense promise in understanding clinical heterogeneity of these syndromes and associated neurocognitive mechanisms.

This study aimed to capture the spectrum of cognitive–behavioural features present across bvFTD and SD, considered as a whole, using data-driven, orthogonally rotated principal component analysis (PCA). When used in a transdiagnostic manner, this method models statistical co-dependencies within and between bvFTD and SD cognitive–behavioural performance data to output a set of core factors or ‘dimensions’. Dimensions are heavily informed by inter-/intra-group performance variations and reflect the involvement of different underlying cognitive processes explaining symptomatic heterogeneity in these syndromes, free from the constraints of categorical labels. By associating these variations to underlying brain network changes, we can further uncover common neural signatures of shared symptomatology in clinically distinct groups. Together, this approach allows us to move *beyond* thinking of heterogeneous and atypical presentations as descriptive of a specific subgroup or a subtype of FTD. Instead, we can capture variations occurring commonly *and* uniquely in both groups, allowing us to position bvFTD and SD patients as varying along a continuous, multidimensional FTD space of cognitive–behavioural changes and associated brain dysfunction.

## Materials and methods

### Participants

Ninety-two participants were recruited through FRONTIER, the frontotemporal dementia research group at the Brain and Mind Centre, The University of Sydney, Australia. Thirty-one patients with a clinical diagnosis of probable bvFTD^38^ and 31 patients with a clinical diagnosis of SD^39^ were included. The SD group was further classified into ‘SD-Left’ (*N* = 20) or ‘SD-Right’ (*N* = 11) based on the magnitude and laterality of ATL and temporopolar atrophy on structural MRI (Supplementary Fig. 1). Unless subgroups are explicitly mentioned, we considered the SD group as one heterogeneous cohort in the analyses.

Diagnoses were established by consensus among a multidisciplinary team comprising senior neurologists (R.M.A. and J.R.H), clinical neuropsychologists and occupational therapists based on comprehensive clinical, neuropsychological and structural MRI assessments. Disease severity in all patients was established using the Clinical Dementia Rating—Frontotemporal Lobar Degeneration Sum of Boxes score (CDR-FTLD SoB^40^), with the frequency of these scores by disease group displayed in Supplementary Fig. 2. Carer ratings on the Cambridge Behavioural Inventory—Revised (CBI-R^41^) and the Neuropsychiatric Inventory (NPI^42^) were used to index the frequency and severity of behavioural changes in patients.

Thirty healthy Control participants, comparable to patient groups for sex and education, were selected through the research volunteer panel and local community clubs. All Controls scored zero on the CDR-FTLD SoB measure and 88 or above on the Addenbrooke’s Cognitive Examination—Revised (ACE-R^43^)—a global assessment of cognitive function spanning attention, memory, verbal fluency, language and visuospatial processing domains. Exclusion criteria for all participants included the history of significant head injury, cerebrovascular disease, alcohol and drug abuse, other primary psychiatric, neurological or mood disorders, and limited English proficiency.

All participants or their person responsible provided written informed consent in accordance with the Declaration of Helsinki. This study was approved by the South Eastern Sydney Local Health District and the University of New South Wales ethics committees.

### General and targeted neuropsychological assessment

Participants underwent comprehensive neuropsychological testing of memory, language and executive functioning (description, scoring and relevant references detailed in Supplementary Methods). Briefly, targeted language assessments of single word naming, comprehension, repetition, semantic association (subtests of the Sydney Language Battery or SYDBAT), as well as auditory attention and working memory (forward and backward scales of the Digit Span Task), and tests of controlled word generation (letter fluency) were included. We also measured performance on visuo-construction abilities and visuospatial recall functions (Rey–Osterrieth Complex Figure or ROCF), executive functions (time difference between parts B and A of Trail Making Test or TMT B–A), as well as an emotion recognition and affect selection task (total score on Facial Affect Selection Task or FAST).

### Behavioural assessments

Carer-rated changes on all subscales of the CBI-R that assess alterations in activities of memory, everyday living, self-care, abnormal behaviours, mood, abnormal beliefs, eating, sleep, stereotypical behaviours and motivation were employed. Clinician-rated changes in agitation, depression, anxiety, apathy, disinhibition and irritability/lability scales from the NPI were assessed (using frequency × severity scores). We note that we did not include other subscales from the NPI (e.g. Delusions, Hallucinations, Euphoria/Elation, Aberrant Motor Behaviour, Night-time Behaviour and Appetite/Eating) to avoid redundancy, as changes in these domains are already captured by the CBI-R. Finally, carer burden was assessed using the Zarit Burden Interview.^44^

### Statistical analyses

Behavioural data were analysed using RStudio v4.0.0^45^ and MATLAB (The Mathworks Inc., Natick, MA, USA).

Prior to describing our analysis pipeline, we clarify our motivation for treating all patients as one heterogeneous FTD cohort for our PCA and neuroimaging analysis but interpreting findings in both a transdiagnostic and syndrome-specific manner. Decoding the magnitude and patterns of cognitive heterogeneity between diagnostic entities requires capturing of both shared and unique performance variance. This was the motivation behind our decision to treat all patient groups as one heterogeneous FTD cohort in our PCA and PCA–neuroimaging correlational analyses. In parallel, recognizing that specific cognitive disturbances may show increased prevalence/magnitude in one specific disease group holds important implications for clinical characterization and decision-making. Therefore, we also conducted and discussed all descriptive analyses, comparisons of PCA scores and visualizations while retaining the clinico-anatomical distinction of SD-Left and SD-Right. In short, combining transdiagnostic and syndrome-specific interpretations of results aided charting of heterogeneous cognitive profiles unique to each clinical group and mapping the prevalence/magnitude of features cutting across syndromic boundaries. For brevity, results from a number of these analyses are described in Supplementary Information.

#### Step 1: characterizing group differences

χ^2^ tests were used to investigate group differences for categorical variables (i.e. sex). For continuous variables, Shapiro–Wilk tests and box-and-whisker plots were first used to determine the normality of distribution. When data met normality assumptions, *t*-tests or ANOVA were used followed by Sidak *post hoc* comparisons. Wilcoxon–Mann–Whitney tests were employed when data violated normality assumptions. For all analyses of group differences, an alpha of *P* ≤ 0.05 was employed.

#### Step 2: standardizing scores and imputing missing data

All subsequent analyses were run in the patient cohort only (*N* = 62). PCA solutions rely on standardized ‘full’ datasets with no missing variables; therefore, missing data were imputed. The combined patient cohort had 4.17% missing data (Supplementary Fig. 3). Available data were converted into percentages following which missing data were imputed using probabilistic PCA with *k*-fold cross-validation (detailed in Supplementary Methods).

#### Step 3: identifying principal cognitive factors using PCA

To determine principal factors of cognitive and behavioural performance, an omnibus PCA employing orthogonal (varimax) rotation was undertaken. Orthogonal rotations maximize dispersion of loadings between components, allow for little shared variance between emergent components, and facilitate clear behavioural and cognitive interpretations. As per recommended approaches,^46^ components with eigenvalues >1.0 were extracted and assigned labels to reflect the majority of variables loading heavily (>|0.5|) on each component.

At the outset, we clarify that factor names are simply shorthand for functions assessed by the majority of tests loading onto that particular factor and, by no means, reflect the entirety of cognitive or behavioural processes underpinning performance across all variables that belong to that factor.

Individual scores on each emergent factor were extracted and used as orthogonal covariates in subsequent behavioural and neuroimaging correlation analyses. To understand patient factor performance in relation to Control performance, we further projected the lower bound of normality score (−1.96 SEM) from Control data into the patient’s PCA space (Supplementary Methods). Group differences between patients on factor scores were examined using ANOVAs with Sidak corrections for *post hoc* comparisons. Finally, associations between factor scores and disease duration were examined using Pearson’s correlations (corrected for multiple comparisons via false discovery rate) in the overall FTD group.

### Image acquisition

Eighty-nine participants (29 bvFTD, 30 SD and 30 Controls) underwent structural MRI using a 3 T Philips MRI scanner with a standard quadrature head coil (eight channels). Whole-brain T_1_-weighted images were acquired using the following parameters: coronal acquisition, matrix 256 × 256 mm, 200 slices, voxel size = 1 mm^3^, echo time/repetition = 2.6/5.8 ms, flip angle *α* = 8°.

### Voxel-based morphometry analyses

Changes in grey matter intensity between groups were investigated using whole-brain voxel-based morphometry (VBM) analyses in FSL (FMRIB Software Library: https://fsl.fmrib.ox.ac.uk/fsl/fslwiki). Pre-processing included brain extraction,^47^ tissue segmentation^48^ and alignment of segmented images to the Montreal Neurological Institute (MNI) standard space using non-linear re-registration.^49,50^ Full details of pre-processing steps are detailed in Supplementary Methods.

#### Whole-brain changes in grey matter intensity

Whole-brain voxel-wise differences in grey matter intensity between bvFTD, SD (whole group as well as SD subgroups), and Control groups were examined using independent *t*-tests with age included as a nuisance variable. Clusters were extracted using the Threshold-Free Cluster Enhancement method using a threshold of *P* < 0.01 corrected for Family-Wise Error (FWE) with a cluster threshold of 100 spatially contiguous voxels.

#### Inter-subject variance in the magnitude and asymmetry of atrophy in disease-specific epicentres

Prior to decomposing cognitive heterogeneity in FTD patients, it is essential to quantify variations in prefrontal and temporal integrity at the individual level, allowing us to position each patient into a continuous frontotemporal atrophy space. Towards this, we calculated the magnitude and asymmetry of atrophy to key frontal and temporal regions that represent potential epicentres of atrophy in bvFTD and SD, respectively.^9,51,52^ We first selected four regions of interest (ROI) in prefrontal [left and right orbitofrontal cortex (OFC)], anterior insula and temporal cortices (left- and right-temporal poles and ATL) from established atlases based on *a priori* knowledge of atrophy epicentres in bvFTD and SD (Supplementary Methods). The ATL masks excluded the temporal poles, so when used in conjunction with temporal pole masks, it allowed us to capture gradation of atrophy along the longitudinal axis of the temporal neocortex (Supplementary Fig. 4). For all patients, mean intensity values for each ROI were extracted and *z*-scored relative to the Control group. Then, two indices for each ROI were computed: (i) a ‘magnitude of atrophy’ index (sum of left and right values), capturing the total amount of atrophy relative to Controls, with smaller numbers indicating greater total bilateral atrophy, and (ii) an ‘asymmetry of atrophy’ index (subtracting values of right from the left ROI) where negative scores indicate left-lateralized atrophy, positive scores indicate right-lateralized atrophy, and scores at/around zero indicate no particularly marked lateralization of atrophy. Group differences were examined on the magnitude score but not on the asymmetry score as it does not index better/worse performance, rather laterality of atrophy.

Complementing this analysis, we computed whole-brain voxel-level inter-subject variance in grey matter intensity to derive brain regions showing uniformly low voxel-level variance. This analysis aids the interpretation of VBM correlation findings, as regions with uniformly low voxel-level variance across cases (coupled with low variation in test scores) may not emerge in VBM correlation analyses, despite their importance in explaining the cognitive–behavioural profile of patients.^32^

#### Correlations of grey matter intensity with PCA-generated factor scores

Finally, VBM correlation analyses were run in the patient group to examine relationships between whole-brain grey matter intensity and performance on emergent PCA factors. A covariate-only statistical model with a positive *t*-contrast was employed with age included as a nuisance variable. We note that for our ‘Behavioural changes’ factor (Factor 1), we found multiple high-loading measures in *both* positive and negative directions; therefore, for this factor, we employed an additional correlation model with a negative *t*-contrast (with age as a nuisance variable). This step is in accordance with previous studies employing two-tailed PCA–VBM correlations specifically for factors comprising measures that load bidirectionally.^9^ Anatomical locations of statistical significance were overlaid on the MNI standard brain with maximum co-ordinates provided in the MNI stereotaxic space. Clusters were extracted using the voxel-wise method with a strict threshold of *P* < 0.001 uncorrected for multiple comparisons with a cluster threshold of 50 spatially contiguous voxels to capture changes in subcortical regions that may relate to emergent factors from the PCA.

## Results

### Demographic, clinical, neuropsychological and behavioural performance

BvFTD patients were significantly younger than Controls (*P* = 0.0009). No other significant group differences emerged for demographic factors (all *P* > 0.1) (Table 1; bvFTD versus SD subgroup findings in Supplementary Results and Supplementary Table 3). Turning to disease severity, irrespective of the diagnostic group, the majority of patients were at very mild-to-mild and moderate stages of disease severity (Supplementary Fig. 2). Importantly, patient groups displayed comparable ages of disease onset, age at diagnosis, disease duration and clinician-indexed disease severity (all *P* > 0.1). Patient groups also displayed significant general cognitive impairment (ACE-R Total) relative to Controls (*P* < 0.001); however, they performed comparably to each other on this measure (*P* = 0.1). Relative to Controls, bvFTD and SD groups also displayed significant impairments on targeted measures of language, attention and working memory, verbal fluency and emotion recognition and affect selection functions (all *P* < 0.01) (Table 2). In addition, executive (TMT B–A) and visuospatial (ACE-R Visuospatial Total and ROCF) dysfunction were evident in bvFTD (all *P* < 0.05).

**Table 1:** Demographic and clinical assessment performance for all groups

	bvFTD	SD	Control	Group effect	bvFTD versus SD (*P-*value)
*N*	31	31	30		
Sex (F: M)	8:23	13:18	15:15	*χ* ^2^ = 3.9; *P* = 0.14	
Age (years)	63.4 (6)	66.2 (7)	69 (5.9)	*F*(2,89) = 5.8; ***P* = 0.004**; $\eta_{p}^{2}$=0.11	0.15
Education (years)	13.9 (2)	13.1 (2.9)	12.9 (2.6)	*F*(2,89) = 1.3; *P* = 0.27; $\eta_{p}^{2}$=0.02	0.18
Age of disease onset (years)	54.6 (6.5)	56.5 (6.5)	–	*t* = −1.03; *P* = 0.30	0.30
Age at diagnosis (years)	58.5 (5.8)	60.9 (6.7)	–	*t* = −1.42; *P* = 0.15	0.15
Disease duration (years)	8.8 (3.6)	9.6 (3.5)	–	*t* = −0.85; *P* = 0.39	0.39
Disease severity (CDR-FTLD SoB)	7.7 (4.5)	6.6 (4.6)	–	*W* = 455; *P* = 0.29	0.29
ACE-R Total (100)	74.7 (18.3)	67 (14.5)	94.9 (2.8)	*F*(2,88) = 33.8; ***P* < 0.001**; $\eta_{p}^{2}$=0.43	0.10

*Note*. In left-most column, maximum test scores reported in brackets; SD group comprises of SD-Left and SD-Right patients (see Supplementary Table 3 for comparisons between these clinical subgroups and bvFTD); for all groups, mean and standard deviation reported; *χ*^2^ = Chi-square value; based on Shapiro–Wilk test outputs, *t*-test (*t*-value) employed when data met normality assumptions or the Wilcoxon–Mann–Whitney test (*W*-value) employed when data violated normality assumptions; for magnitude of group effect, exact *χ*^2^/*t-*/*W*-/*F-*statistics, exact *P*-values (unless *P* < 0.001) and effect size ($\eta_{p}^{2}$) values reported; for all statistical comparisons, *P*-values bolded if *P* ≤ 0.05; bvFTD, behavioural variant frontotemporal dementia; SD, semantic dementia; CDR-FTLD SoB, Clinical Dementia Rating—Frontotemporal Lobar Degeneration Sum of Boxes; ACE-R, Addenbrooke’s Cognitive Examination—Revised.

**Table 2:** Neuropsychological assessment performance for all groups

	bvFTD	SD	Control	Group effect	bvFTD versus SD (*P-*value)
ACE-R attention Total (18)	15 (3.8)	16 (1.7)	17.5 (0.6)	*F*(2,88) = 8.1; *P* < **0.001**; $\eta_{p}^{2}$=0.15	0.77
ACE-R memory Total (26)	17.3 (6.3)	15 (5.3)	24.4 (1.7)	*F*(2,88) = 29.8; *P* < **0.001**; $\eta_{p}^{2}$=0.40	0.21
ACE-R fluency Total (14)	6.8 (4.4)	6.2 (3.2)	12.2 (1.5)	*F*(2,88) = 30.6; *P* < **0.001**; $\eta_{p}^{2}$=0.41	0.54
ACE-R language Total (26)	21.6 (4.4)	14.7 (4.7)	25.3 (0.99)	*F*(2,88) = 62; *P* < **0.001**; $\eta_{p}^{2}$=0.58	0.**0001**
ACE-R visuospatial Total (16)	13.9 (2.4)	14.9 (2.2)	15.3 (1)	*F*(2,88) = 3.5; *P* = **0.032**; $\eta_{p}^{2}$=0.07	0.058
SYDBAT Naming (30)	22.4 (5.1)	9 (6.1)	27 (1.8)	*F*(2,84) = 112.6; *P* < **0.001**; $\eta_{p}^{2}$=0.72	**<0**.**0001**
SYDBAT Comprehension (30)	26.7 (2.8)	20.6 (6.8)	29.2 (1.5)	*F*(2,86) = 29.9; *P* < **0.001**; $\eta_{p}^{2}$=0.41	0.**0018**
SYDBAT Repetition (30)	29.4 (1.2)	29 (1.7)	29.8 (0.4)	*F*(2,85) = 2.8; *P* = 0.06; $\eta_{p}^{2}$=0.07	0.0603
SYDBAT Semantic Association (30)	24.3 (4.1)	18.9 (6)	28.3 (1.6)	*F*(2,85) = 35.1; *P* < **0.001**; $\eta_{p}^{2}$=0.45	0.**0029**
Digit span forward (16)	9.1 (2.1)	10.1 (2.4)	11.8 (2)	*F*(2,88) = 10.6; *P* < **0.001**; $\eta_{p}^{2}$=0.19	0.20
Digit span backward (16)	5.3 (2.2)	6.4 (2.2)	7.8 (2.3)	*F*(2,88) = 9.3; *P* < **0.001**; $\eta_{p}^{2}$=0.17	0.057
Letter fluency (F, A, S total)	25.1 (16.3)	26 (10.2)	44.6 (10.2)	*F*(2,87) = 22.6; *P* < **0.001**; $\eta_{p}^{2}$=0.34	0.69
ROCF copy (36)	29.2 (5.9)	31.4 (3.1)	32 (2.9)	*F*(2,86) = 3.7; *P* = **0.027**; $\eta_{p}^{2}$=0.08	0.30
ROCF delayed recall (36)	10.3 (7.6)	14.1 (7.1)	17.1 (5.1)	*F*(2,83) = 7.2; *P* = **0.0013**; $\eta_{p}^{2}$=0.14	0.065
TMT B–A Time Difference (s)	78.7 (45.8	68 (76.2)	44.9 (26.5)	*F*(2,80) = 2.7; *P* = 0.072; $\eta_{p}^{2}$=0.06	0.12
FAST total (42)	30.5 (6.9)	30.4 (5.6)	38.7 (2.9)	*F*(2,89) = 22.9; *P* < **0.001**; $\eta_{p}^{2}$=0.34	0.72

*Note*. In left-most column, maximum test scores reported in brackets; SD group comprises of SD-Left and SD-Right patients (see Supplementary Table 3 for comparisons between these clinical subgroups and bvFTD); for all groups, mean and standard deviation reported; *χ*^2^ = Chi-square value; based on Shapiro–Wilk test outputs, *t*-test (*t*-value) employed when data met normality assumptions or Wilcoxon–Mann–Whitney test (*W*-value) employed when data violated normality assumptions; for the magnitude of group effect, exact *χ*^2^/*t-*/*W*-/*F-*statistics, exact *P*-values (unless *P* < 0.001) and effect size ($\eta_{p}^{2}$) values reported; for all statistical comparisons, *P*-values bolded if *P* ≤ 0.05; bvFTD, behavioural variant frontotemporal dementia; SD, semantic dementia; ACE-R, Addenbrooke’s Cognitive Examination—Revised; SYDBAT, Sydney Language Battery; ROCF, Rey–Osterrieth Complex Figure; TMT B–A, Trail Making Test parts B–A; FAST, Facial Affect Selection Test.

A direct comparison between bvFTD and the overall SD group revealed no significant differences across screening measures of attention, memory, fluency and visuospatial functions, and targeted tests of repetition, auditory attention and working memory, letter fluency, visuospatial, executive and emotion processing (Table 2). These findings provide preliminary evidence, at the group level, for pervasive overlap in cognitive dysfunction in these syndromes despite comparable disease staging. Compared with bvFTD, SD patients performed poorly on global language assessments, as well as on targeted measures of naming, comprehension and semantic association (all *P* < 0.005). Turning to behavioural measures, many carer-rated behavioural disturbances were not found to differ significantly between bvFTD and SD (Table 3; all *P* > 0.059); however, apathy, motivational changes, self-care difficulties, eating and sleeping abnormalities were rated as more severe in bvFTD relative to SD (all *P* < 0.05). Carers of bvFTD patients further reported significantly greater burden relative to SD carers (*P* = 0.044).

**Table 3:** Behavioural assessment performance for all patient groups

	bvFTD	SD	Group effect	bvFTD versus SD (*P-*value)
NPI Agitation (12)	2.3 (2.6)	0.9 (1.3)	*W* = 514; *P* = 0.068	0.068
NPI Depression (12)	1.3 (2.9)	1.9 (2.2)	*W* = 265.5; *P* = 0.059	0.059
NPI Anxiety (12)	1.1 (2.6)	0.96 (1.5)	*W* = 353; *P* = 0.63	0.63
NPI Apathy (12)	5.1 (3.9)	2 (2.9)	*W* = 584; ***P* = 0.003**	0.**003**
NPI Disinhibition (12)	3.2 (3.9)	1.6 (2.6)	*W* = 484.5; *P* = 0.11	0.11
NPI Irritability (12)	1.9 (2.7)	0.88 (1.5)	*W* = 441; *P* = 0.23	0.23
CBI-R Memory (%)	41.1 (19.8)	40.4 (18.6)	*t* = 0.13; *P* = 0.88	0.88
CBI-R Everyday skills (%)	28.1 (26.2)	18.1 (22.3)	*t* = 1.5; *P* = 0.11	0.11
CBI-R Self-Care (%)	15.3 (26.5)	2.9 (7.6)	*t* = 2.5; ***P* = 0.014**	0.**014**
CBI-R Abnormal behaviour (%)	31.7 (19.2)	25 (27.2)	*t* = 1.1; *P* = 0.27	0.27
CBI-R Mood (%)	25 (20.1)	25 (21)	*t* = 0; *P* = 1	1
CBI-R Abnormal beliefs (%)	6.9 (18.5)	5.5 (10.7)	*t* = 0.3; *P* = 0.71	0.71
CBI-R Eating changes (%)	40.1 (31.5)	21.4 (25)	*t* = 2.5; ***P* = 0.013**	0.**013**
CBI-R Sleep changes (%)	40.7 (30.7)	23.3 (22.6)	*t* = 2.5; ***P* = 0.014**	0.**014**
CBI-R Stereotypical behaviour (%)	47.3 (28.2)	38.1 (32.2)	*t* = 1.1; *P* = 0.23	0.23
CBI-R Motivational changes (%)	54 (33.9)	37.1 (30.5)	*t* = 2; ***P* = 0.045**	0.**045**
ZBI Total (88)	22.6 (9.7)	17.5 (9.4)	*t* = 2.05; ***P* = 0.044**	0.**044**

*Note*. In left-most column, maximum test scores reported in brackets; SD group comprises of SD-Left and SD-Right patients (see Supplementary Table 3 for comparisons between these clinical subgroups and bvFTD); for all groups, mean and standard deviation reported; *χ*^2^ = Chi-square value; based on Shapiro–Wilk test outputs, *t*-test (*t*-value) employed when data met normality assumptions or Wilcoxon–Mann–Whitney test (*W*-value) employed when data violated normality assumptions; for the magnitude of group effect, exact *χ*^2^/*t-*/*W*-/*F-*statistics, exact *P*-values (unless *P* < 0.001) and effect size ($\eta_{p}^{2}$) values reported; for all statistical comparisons, *P*-values bolded if *P* ≤ 0.05; bvFTD, behavioural variant frontotemporal dementia; SD, semantic dementia; NPI, Neuropsychiatric Inventory; ZBI, Zarit Burden Interview; CBI-R, Cambridge Behavioural Inventory—Revised.

### Patterns of whole-brain grey matter atrophy

Next, we present results from whole-brain/ROI grey matter atrophy and variance analyses as these provide a snapshot of co-occurring frontotemporal degeneration in these groups. These findings are used as a foundation to explore transdiagnostic patterns of cognitive–behavioural variation arising from degeneration of common brain systems in these syndromes.

Relative to Controls, bvFTD patients displayed reduced grey matter intensity centred on bilateral lateral and medial prefrontal cortices, insula and OFC, extending towards temporal poles and ATL, medial temporal and striatal regions, and laterally to lateral temporal, posterior parietal and occipital and cerebellar regions (Supplementary Table 1 and Supplementary Fig. 5). Relative to Controls, SD patients displayed reduced grey matter intensity centred on the bilateral temporal poles and ATL, extending dorsally into bilateral OFC, insula and frontal poles, and medially into medial temporal and posterior parietal regions. Similar patterns were observed when comparing SD subgroups with Controls, albeit largely lateralized to the left hemisphere for SD-Left and right hemisphere for SD-Right groups (Supplementary Table 2). Comparing patient groups, bvFTD patients displayed greater atrophy to bilateral prefrontal and anterior cingulate cortices compared with SD, while SD patients displayed greater atrophy to bilateral lateral and medial temporal cortices compared with the bvFTD group. Comparing SD subgroups with each other, SD-Right displayed greater atrophy to right anterior, medial and posterior temporal regions, extending into right ventral occipital and inferior parietal cortices, whereas no significant clusters emerged for the reverse contrast (see Supplementary Table 2). In the final set of comparisons, we examined differences in atrophy patterns between bvFTD and SD subgroups. Overall, bvFTD displayed significantly greater prefrontal atrophy compared with both SD subgroups. Relative to the SD-Left group, greater right-lateralized prefrontal atrophy was noted in bvFTD, whereas the inverse pattern (i.e. greater left-lateralized prefrontal and insular involvement) was noted when compared to SD-Right. Comparing SD subgroups with bvFTD, greater temporal involvement, lateralized by diagnostic category, was noted in SD subgroups.

### Whole-brain voxel-level inter-subject grey matter variance

Visual inspection of grey matter intensity variance maps revealed uniformly low voxel-level variance in bilateral prefrontal (OFC), insula, ATL, posterior temporal, striatal and subcortical, parietal midline and cerebellar regions. These findings align with our VBM atrophy analyses as many of these regions represent sites of maximal atrophy in our bvFTD and SD groups, whereas regions flanking the ‘edges’ of atrophy clusters show greater grey matter intensity variance (Supplementary Fig. 5).

### Inter-subject variance in atrophy to disease-specific epicentres

#### Magnitude of atrophy

**Figure 1 Variance in the magnitude and asymmetry of atrophy in bvFTD and SD in disease-specific epicentres.**
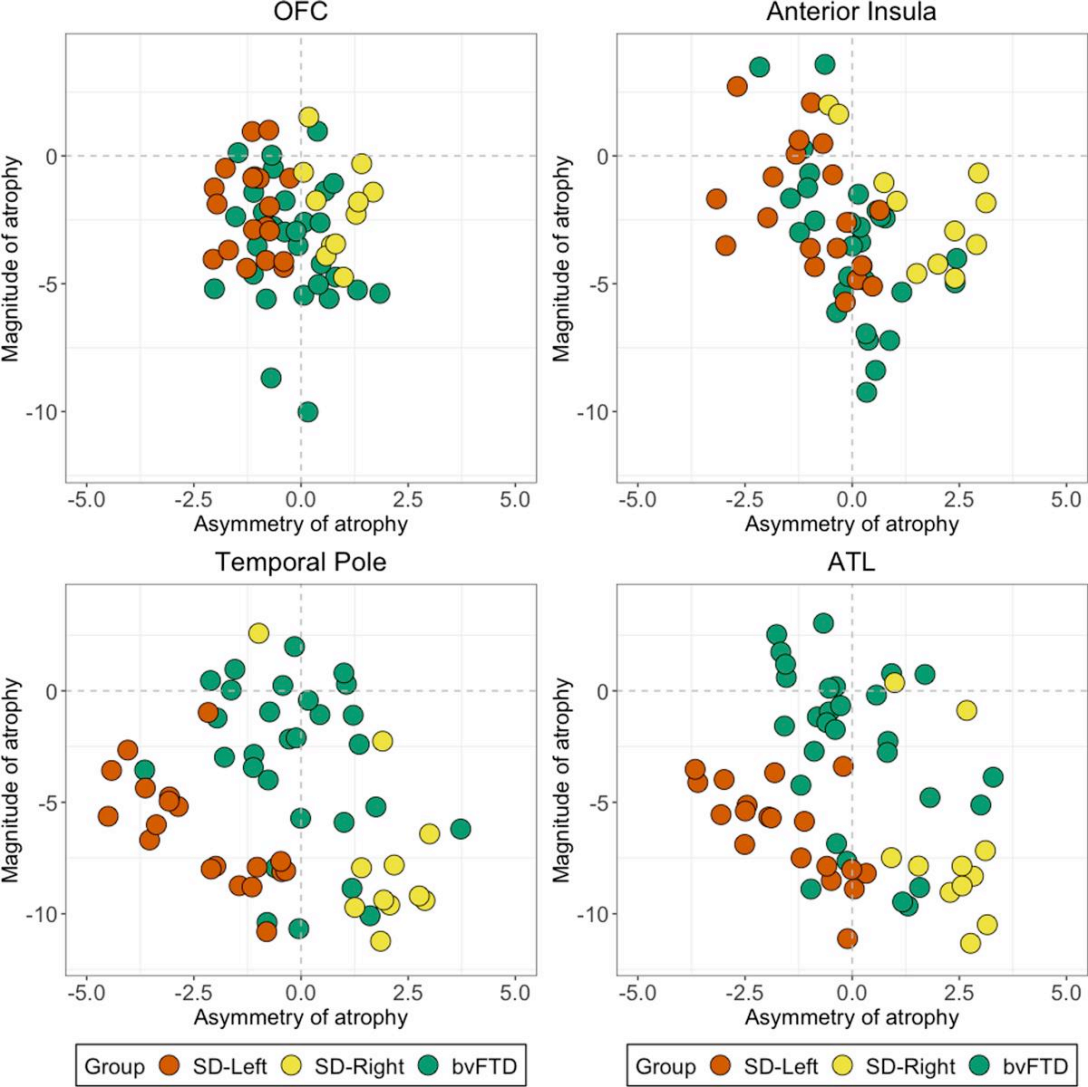
All scores are *z*-scored (relative to the Control group) mean intensity values for *a priori* bi-hemispheric regions of interest. The magnitude index (i.e. left + right intensity values) captures the total amount of atrophy relative to Controls, and the asymmetric index (i.e. left–right score) captures asymmetry of atrophy. For the magnitude index, analyses of variance indicated significantly greater atrophy in the temporal pole [*F*(2,56) = 7.8; *P* < 0.001; $\eta_{p}^{2}$ = 0.21] and ATL [*F*(2,56) = 11.3; *P* < 0.001; $\eta_{p}^{2}$ = 0.28] in both SD groups relative to the bvFTD group. In contrast, no significant differences emerged for the OFC [*F*(2,56) = 2.9; *P* = 0.060; $\eta_{p}^{2}$ = 0.09] and anterior insula [*F*(2,56) = 2.07; *P* = 0.13; $\eta_{p}^{2}$ = 0.06] magnitude indices (further details in Supplementary Table 3). On the asymmetry index, negative scores suggest left-lateralized atrophy, positive scores suggest right-lateralized atrophy and scores at/close to zero indicate no particularly marked lateralization of atrophy. No statistical comparisons were conducted for asymmetry analyses (i.e. left–right) as the outcome index does not measure better or worse performance but rather the laterality of atrophy. bvFTD, behavioural variant frontotemporal dementia; SD, semantic dementia.

BvFTD, SD-Left and SD-Right patients demonstrated comparable magnitude of atrophy with bilateral OFC and anterior insular cortices (all *P* > 0.06; Fig. 1 and Supplementary Table 3). On scatterplots, this is visible as dense overlap between bvFTD and SD patients, especially for OFC integrity (Fig. 1). For temporal regions, in contrast, the magnitude of ATL/temporal pole atrophy was significantly greater in SD (and in both SD subgroups) compared with bvFTD patients (all *P* < 0.001; Fig. 1 and Supplementary Table 3).

#### Lateralization of atrophy

For prefrontal cortex ROIs, the bvFTD group displayed evenly distributed atrophy in left and right OFC and anterior insular cortices. For temporal regions, the bvFTD group displayed relatively even bi-hemispheric atrophy in temporal poles, while most patients tended to display left-lateralized ATL involvement (Fig. 1). For SD, in contrast, atrophy in both prefrontal and temporal regions was lateralized greatly to the hemisphere primarily affected in their individual SD subgroup diagnosis (Fig. 1).

### Determining principal factors underlying cognitive–behavioural performance

Emergent factors and test loadings from the PCA are displayed in Fig. 2 and Supplementary Table 4. The sample size was considered adequate for PCA (Kaiser–Meyer–Olkin statistic = 0.69). Both probabilistic and varimax-rotated PCAs converged on a solution with eight orthogonal components with eigenvalues >1, together explaining 73.4% of the performance variance in the patient cohort (Supplementary Figs. 6 and 7).

**Figure 2 Factor loadings for neuropsychological and behavioural measures in the combined behavioural variant frontotemporal dementia and semantic dementia group (N = 62) on a varimax-rotated 8 component PCA solution.**
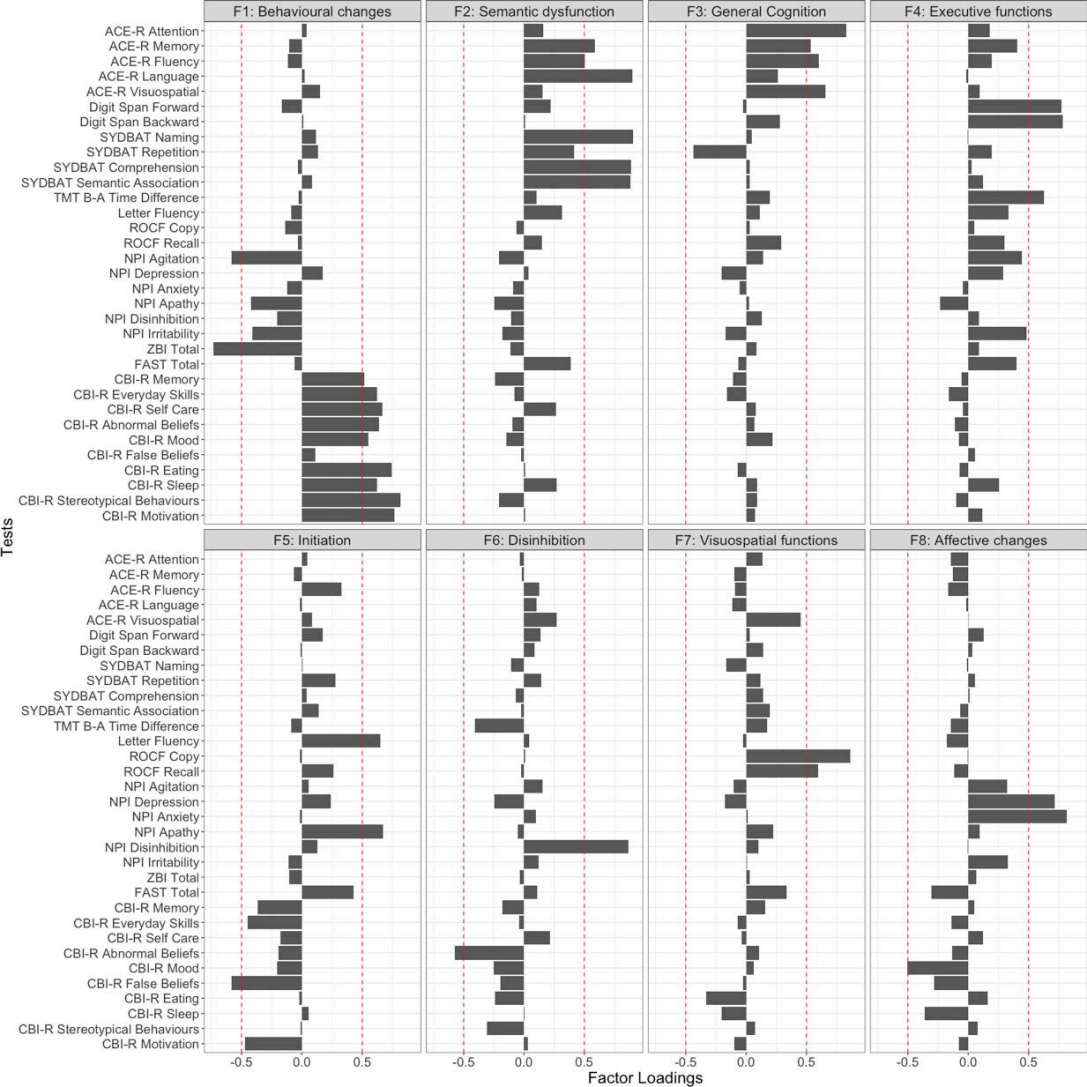
Panel indicates emergent factors, in the order of the amount of overall variance explained. Red dashed lines representing factor loading cut-offs (>|0.5|). The varimax-rotated PCA converged on a solution with eight orthogonal components with eigenvalues >1, together explaining 73.4% of the performance variance in the patient cohort (component-specific variance explained for Factor 1 = 21.5%; Factor 2 = 18.5%; Factor 3 = 8.5%; Factor 4 = 6.9%; Factor 5 = 5.4%; Factor 6 = 4.6%; Factor 7 = 3.9%; Factor 8 = 3.8%). ACE-R, Addenbrooke’s Cognitive Examination—Revised; SYDBAT, Sydney Language Battery; TMT B–A, Trail Making Test parts B–A; ROCF, Rey–Osterrieth Complex Figure; NPI, Neuropsychiatric Inventory; ZBI, Zarit Burden Interview; FAST, Facial Affect Selection Task; CBI-R, Cambridge Behavioural Inventory—Revised.

Factor 1 was labelled ‘Behavioural changes’, explained 21.5% of the overall variance and loaded positively on measures of behavioural changes (CBI-R stereotypical and abnormal behaviour, motivation, eating, self-care and everyday skills, sleep, mood and memory subscales) and negatively with the NPI Agitation score and overall carer burden (ZBI Total), suggesting that patients with more preserved everyday behaviours (e.g. motivation, sleep, self-care and mood) were less likely to be agitated and were associated with less carer burden.

Factor 2 was titled ‘Semantic dysfunction’, captured 18.5% of the overall variance and loaded mainly on tasks of semantic functions such as SYDBAT Naming, Comprehension and Semantic Association subtests, and ACE-R Language, Memory and Fluency subscales.

Factors 3 and 4 accounted for 8.5 and 6.9% of the total variance, respectively. Tests loading on Factor 3 included ACE-R Memory, Fluency, Attention and Visuospatial subscales and was labelled ‘General cognition’. Factor 4 was named ‘Executive functions’ and included measures of sustained attention and working memory (Digit Span Forward and Backward) and overall executive performance and processing speed (TMT B–A measure).

The fifth and sixth factors, respectively, captured 5.4 and 4.6% of the overall variance. Factor 5 was named ‘Initiation’ and mainly loaded on Letter Fluency and NPI Apathy scores, as well as the CBI-R false belief measure. Factor 6 was referred to as ‘Disinhibition’ as it loaded on the NPI disinhibition and CBI-R abnormal behaviour subscales.

The seventh factor explained 3.9% of the overall variance and was labelled ‘Visuospatial functions’, loading heavily on ROCF Copy and Delayed Recall components. The eighth factor was named ‘Affective changes’, captured 3.8% of the overall variance, and included the CBI-R Mood, and NPI depression and anxiety subscales. Measures not loading heavily on any factors included the SYDBAT Repetition, FAST Total score and the NPI irritability subscore.

#### Graded overlap and differences in performance between bvFTD, SD-Left and SD-Right patients

**Figure 3 Factor scores for bvFTD and SD patients on emergent factors.**
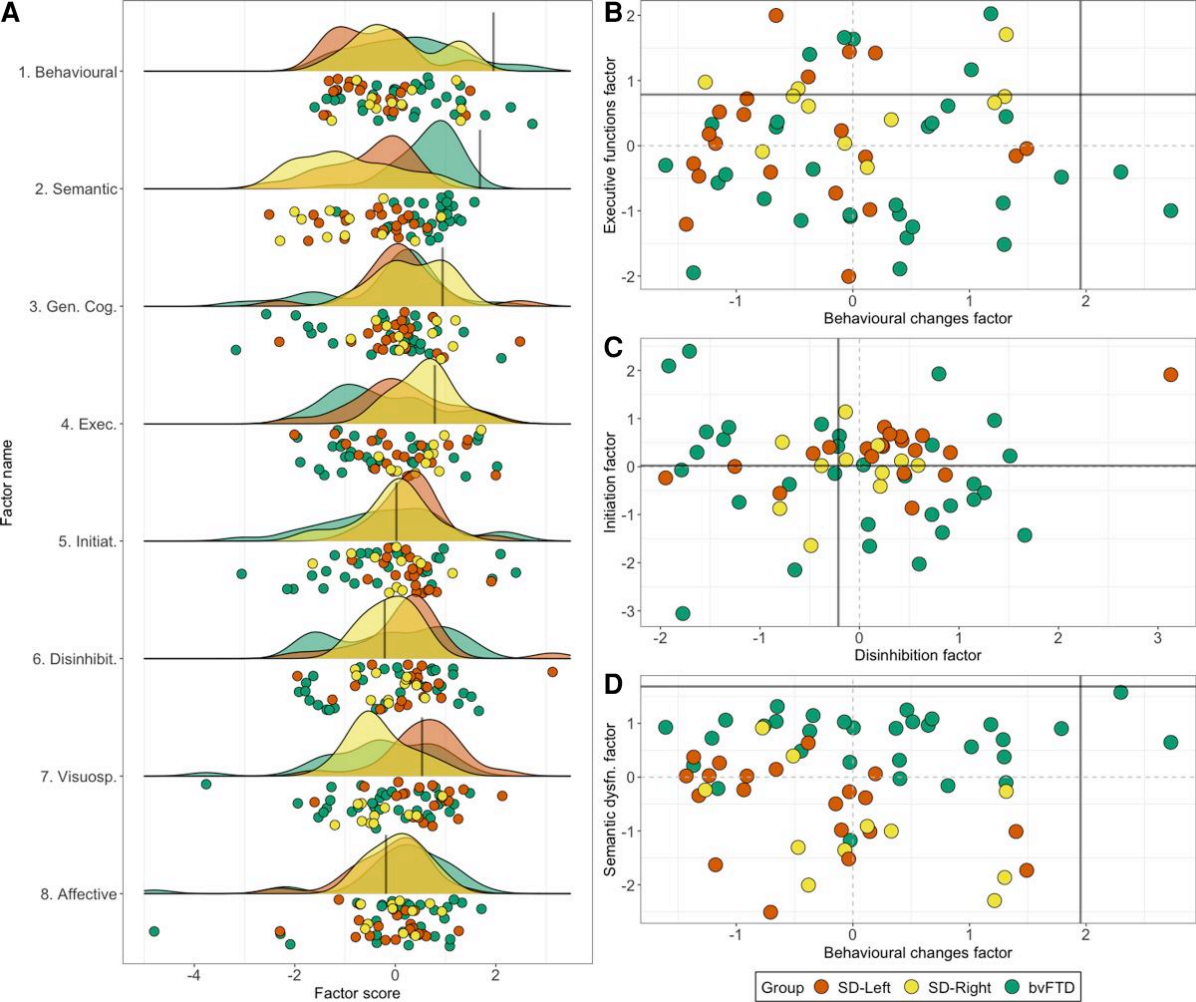
(**A**) Raincloud plots showcasing group-level density of distribution (cloud) and individual jittered points (rain) of performance variation across PCA-derived factors for bvFTD and SD patients. Scatter plots for select factors displaying relationships between (**B**) Behavioural changes (Factor 1) with Executive function (Factor 4), (**C**) Initiation (Factor 5) and Disinhibition (Factor 6) factors, and (**D**) Behavioural changes (Factor 1) and Semantic dysfunction (Factor 2) factors. In all plots, black lines indicate lower bound of normality (−1.96 SEM) as estimated from the Control group (calculation detailed in Supplementary Methods). Positive scores approaching the lower bound of normality indicate better performance. Statistical comparisons run using ANOVA with *post hoc* comparisons using Sidak corrections indicated significant group differences in Semantic dysfunction factor [Factor 2: *F*(2,59) = 25.3; *P* < 0.001; $\eta_{p}^{2}$ = 0.46] with both SD groups performing significantly poorer than the bvFTD group (all *P* < 0.001) but comparably to each other (*P* > 0.1); Executive factor [Factor 4: *F*(2,59) = 3.1; *P* = 0.05; $\eta_{p}^{2}$ = 0.09] where the bvFTD group performed significantly worse than the SD-Right group (*P* = 0.007); and the Visuospatial factor [Factor 7: *F*(2,59) = 5.1; *P* = 0.008; $\eta_{p}^{2}$ = 0.14] where both bvFTD and SD-Right patients performed poorer than the SD-Left group (both *P-*values < 0.01), with no significant differences between bvFTD and SD-Right groups (*P* > 0.1). No other significant differences emerged. bvFTD, behavioural variant frontotemporal dementia; SD, semantic dementia.

**Figure 4 Group differences between bvFTD, SD-Left and SD-Right patients on emergent factors from the PCA.**
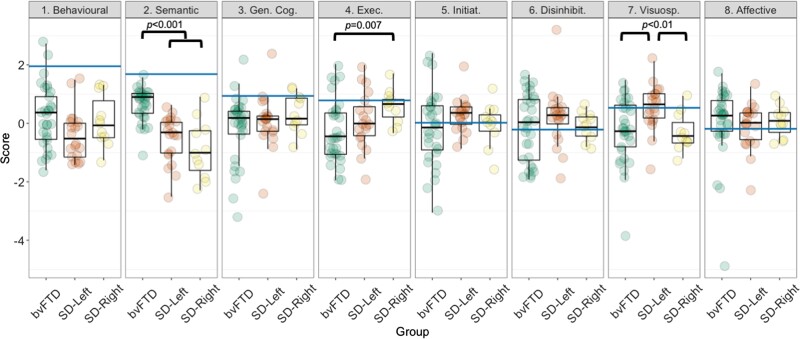
Statistical comparisons run using ANOVA with *post hoc* comparisons using Sidak corrections indicated significant group differences in Semantic dysfunction factor [Factor 2: *F*(2,59) = 25.3; *P* < 0.001; $\eta_{p}^{2}$ = 0.46] with both SD groups performing significantly poorer than the bvFTD group (all *P* < 0.001) but comparably to each other (*P* > 0.1); Executive factor [Factor 4: *F*(2,59) = 3.1; *P* = 0.05; $\eta_{p}^{2}$ = 0.09] where the bvFTD group performed significantly worse than the SD-Right group (*P* = 0.007) and the Visuospatial factor [Factor 7: *F*(2,59) = 5.1; *P* = 0.008; $\eta_{p}^{2}$ = 0.14] where both bvFTD and SD-Right patients performed poorer than the SD-Left group (both *P-*values < 0.01), with no significant differences between bvFTD and SD-Right groups (*P* > 0.1). No other significant differences emerged. All *P*-values emerging as significant in *post hoc* comparisons are indicated in figure. Blue horizontal lines indicate the Control lower bound of normality score (i.e. −1.96 SEM from Control performance; see Supplementary Methods for calculation details). bvFTD, behavioural variant frontotemporal dementia; SD, semantic dementia; PCA, principal component analysis; Gen. Cog., General Cognition factor; Exec., Executive factor; Initiat., Initiation factor; Disinhibit., Disinhibition factor; Visuosp., Visuospatial factor.

Individual-level performance, group-level gradations and scatter plots for select factors are presented in Fig. 3. Group comparisons on factors for bvFTD and SD subgroups are displayed in Fig. 4 (results and figures for group comparisons between bvFTD and the whole SD group can be found in Supplementary Results and Supplementary Fig. 8). Marked overlap across original diagnostic templates was noted (Figs. 3A and 4). On the Semantic dysfunction factor (Factor 2), significant group differences emerged [*F*(2,59) = 25.3; *P* < 0.001; $\eta_{p}^{2}$ = 0.46], whereby both SD groups performed significantly poorer than the bvFTD group (all *P-*values < 0.001), but comparably to each other (*P* > 0.1). Significant group differences also emerged on the Executive factor (Factor 4) [*F*(2,59) = 3.1; *P* = 0.05; $\eta_{p}^{2}$ = 0.09], where the bvFTD group performed significantly worse than the SD-Right group (*P* = 0.007). Finally, on the Visuospatial factor (Factor 7), significant group differences were noted [*F*(2,59) = 5.1; *P* = 0.008; $\eta_{p}^{2}$ = 0.14]. On this factor, both bvFTD and SD-Right patients performed poorer than the SD-Left group (both *P-*values < 0.01), with no significant differences between bvFTD and SD-Right groups (*P* > 0.1). No other significant differences emerged.

### Associations between factor performance and disease duration

In the combined FTD cohort, no significant associations were noted between emergent factor scores and disease duration (all *r-*values <0.29 and >−0.21; all *P-*values >0.1) (Supplementary Table 5).

### Neural correlates of principal cognitive–behavioural factors

**Figure 5 Regions of grey matter intensity correlating with PCA-generated factors in the patient cohort.**
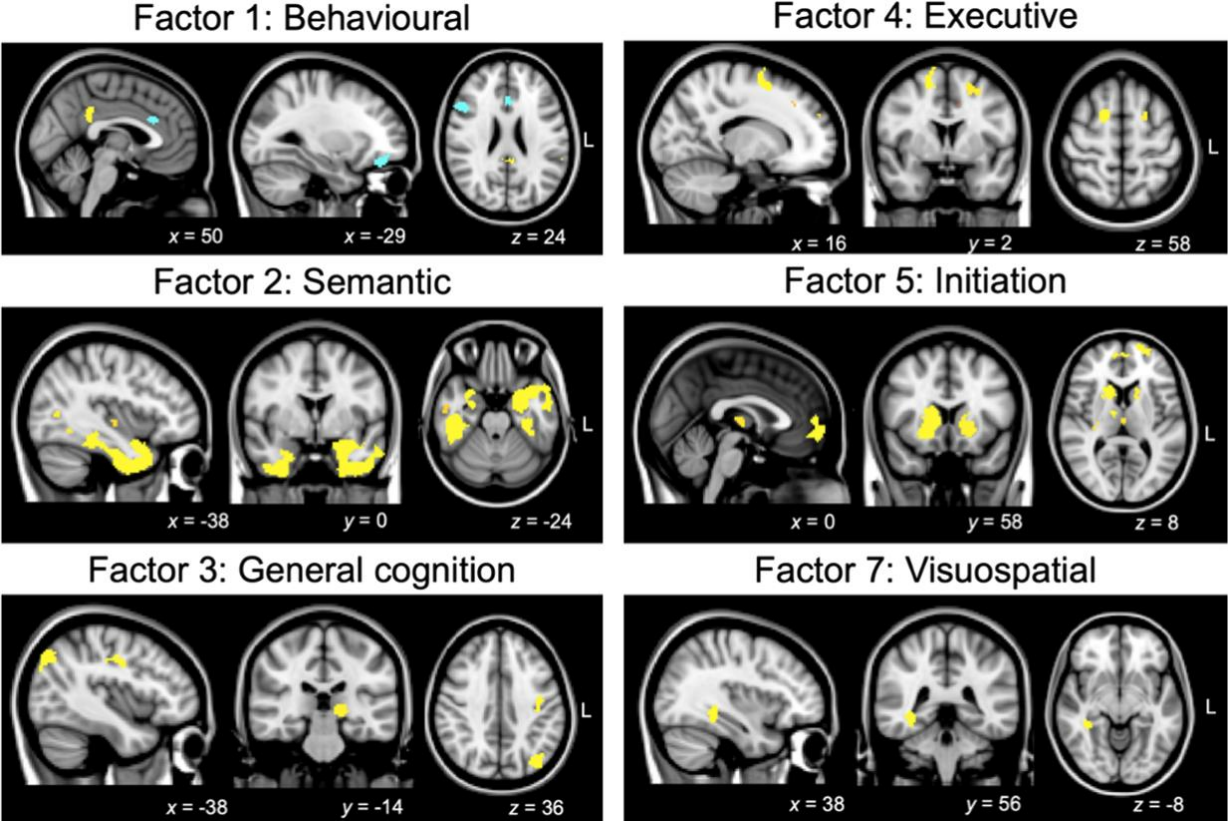
All factors are derived from varimax-rotated PCA of cognitive–behavioural measures in the combined bvFTD-SD group. Statistical associations derived via voxel-based morphometry correlation analysis using a covariate-only statistical model with positive/negative *t*-contrasts, with age included as a nuisance covariate in the analysis. Coloured voxels indicate regions emerging as significant in the voxel-based morphometry analyses at a threshold of *P* < 0.001 uncorrected for multiple comparisons with a cluster threshold of 50 contiguous voxels (yellow denotes positive associations; blue denotes negative associations). All clusters reported at *t* = 3.97 (see Supplementary Table 6 for detailed cluster and *t*-value information). Clusters are overlaid on the Montreal Neurological Institute (MNI) standard brain with *x*, *y* and *z* co-ordinates reported in the MNI standard space. L, left; bvFTD, behavioural variant frontotemporal dementia; SD, semantic dementia; PCA, principal component analysis.

Associations between grey matter intensity and PCA-generated factor scores in patients are displayed in Fig. 5 and Supplementary Table 6. Irrespective of clinical diagnosis, Behavioural changes (Factor 1) was associated with grey matter intensity changes in left OFC, frontal pole and anterior cingulate cortex, right inferior frontal gyrus, left supramarginal gyrus and parietal operculum cortex, and bilateral posterior cingulate cortex and precuneus. Semantic dysfunction (Factor 2) was associated with grey matter intensity in bilateral ATLs and lateral temporal cortices, including bilateral temporal poles extending ventrally into temporal fusiform gyri and medially into the right medial temporal lobe (hippocampus, amygdala and putamen). Regions in the left insular cortices and Heschl’s gyrus, lateral temporal cortices (bilateral middle/inferior temporal gyri) and the left inferior parietal cortex (angular gyrus) also emerged as significant. Performance on the General cognition factor (Factor 3) was associated with left-lateralized occipital, parietal (precentral/postcentral gyrus) and subcortical (left thalamus) regions. For the Executive functions factor (Factor 4), prefrontal regions such as bilateral superior frontal and left middle/inferior frontal gyri, left insular cortex and the left frontal pole emerged as significantly associated with performance. Initiation (Factor 5) difficulties were associated with grey matter alterations in bilateral prefrontal (frontal medial cortex, frontal poles, OFC, superior frontal gyrus and paracingulate gyrus), bilateral subcortical (putamen, caudate, thalamus and left pallidum and nucleus accumbens), as well as right insular cortices. Finally, performance on the Visuospatial factor (Factor 7) was associated with right ventral temporal regions including temporal/temporo-occipital fusiform and lingual gyri as well as the right posterior parahippocampal cortex. These specific regions are not only areas of maximal atrophy in bvFTD and SD but rather flank regions of maximal atrophy and are areas of greater grey matter variance (Supplementary Fig. 5). No significant clusters emerged for Disinhibition (Factor 6) or Affective changes (Factor 8) factors at *P*_unc_ < 0.001.

## Discussion

This study demonstrates marked overlap of cognitive–behavioural performance in a large group of well-characterized patients clinically labelled as bvFTD and those labelled as SD. Using PCA, we identified eight independent, separable dimensions explaining cognitive–behavioural performance variations along which clinical entities of bvFTD and SD were systematically located. Notably, bvFTD and SD showed systematic variation, undifferentiated at a group level, across five of the emergent factors, namely Behavioural changes, General Cognition, Initiation, Disinhibition and Affective changes. Behavioural correlations in the whole FTD group revealed no significant associations between factor scores and disease duration, suggesting that the profiles of cognitive impairment in FTD observed here may emerge independent of advancing disease. Finally, performance on these factors reflected the differential degeneration of fronto-insular, parietal and striatal regions. In contrast, we found evidence of discrete factors that better differentiated between the patient groups. Semantic dysfunction was, unsurprisingly, associated with SD and reflected ATL and temporal lobe atrophy, while Executive and Visuospatial dysfunction were more characteristic of bvFTD, attributable to fronto-insular and ventral temporal atrophy, respectively.

Before discussing our findings and their clinical implications in detail, it is important to pause and understand what our transdiagnostic conceptualization of FTD (here, restricted to bvFTD and SD) represents and what it *does not* mean. Traditional approaches to exploring cognitive–behavioural variations in FTD seek out mutually exclusive diagnostic categories and subcategories. As newer symptoms and their variations are discovered, subcategories continue to grow, with a continuing sequence of studies proposing new subtypes and categories falling under an ‘FTD space’. This pursuit is perfectly reasonable, provided there is sufficient intra-category homogeneity and inter-category separation that is reliably detected across studies, cohorts and techniques. In clinical practice and across independent bodies of evidence,^9,13,34^ however, graded performance variations within and between mutually exclusive categorisations are evident. Much of this variation is closely tied to individual differences in underlying neural degeneration, pathological processes and disease progression.^53^ Our transdiagnostic approach aids the extraction of these systematic performance variations observed across patients and also maps inter-individual variations within a graded FTD space. This approach allows to preserve the well-known separation of prototypical clinical descriptions of ‘pure’ SD and bvFTD as situated in different areas within this space; yet the same model can also accommodate graded patient variations and overlapping symptom dimensions. Dimensions, however, *do not reflect* new categories of symptomatic changes or performance deficits; rather they are axes of the multidimensional space. In accordance with this view, the clinical translation of our findings is that while bvFTD and SD display marked heterogeneity across multiple cognitive–behavioural dimensions, this heterogeneity is *graded* and not absolute between syndromes, and varies at the individual level.

Using whole-brain VBM, we validated our PCA approach by demonstrating discrete neuroanatomical signatures of emergent factors, which are in line with the extant cognitive neuroscience and neurology literature. For example, our neural correlates of Behavioural changes, Initiation and Executive functions resonate with a large body of work implicating bilateral fronto-insular and striatal regions in the origin of these disturbances in FTD.^54–60^ In contrast, Semantic disturbances, as expected, were associated with bilateral temporopolar, ATL and posterior portions of the ATL bordering posterior temporal/inferior parietal regions. Bilateral ATLs form trans-modal hubs of semantic processing in the brain,^6^ the earliest sites of metabolic and structural changes in SD,^61^ with atrophy progressing along the ATL correlating with increasing semantic impairment in FTD.^62^ In the temporal lobe, we further found right ventral temporal regions to correlate with Visuospatial changes in FTD. These regions are important for the integration of complex visuospatial information^63^ and have typically received attention in the SD-Right literature for their potential role in mediating visuospatial and face-processing difficulties.^64^ We note, however, that these ventral temporal sites are not regions of earliest atrophy in bvFTD and SD-Left. Indeed, they showed greater inter-subject grey matter variance, and therefore, possibly displayed increased sensitivity to being detected by our VBM correlation analyses. Finally, posterior parietal cortices and subcortical thalamic areas emerged as associated with performance on the General Cognition factor. Degeneration of the parietal cortex, although typically emerging in later FTD disease stages,^65^ is a key candidate for executive, general cognitive and behavioural dysfunction, as found in investigations of the behavioural/dysexecutive and aphasic variants of Alzheimer’s disease.^35,66,67^ On the other hand, striatal and thalamic structural degeneration are present early in the disease course in FTD^65,68,69^ and may explain a variety of goal-directed disturbances in FTD.^56,70^ Collectively, these results indicate that (i) performance gradations emerge amidst overlapping distributions of frontal and temporal atrophy across bvFTD and SD and (ii) distinct areas of grey matter changes underpin dimensional performance along each respective factor.

It is important to consider these findings with respect to current classification practices of FTD. While the international consensus criteria for bvFTD^38^ and SD^71^ have been invaluable for refining the identification and characterization of FTD patients worldwide, they do not acknowledge the degree of overlap between cases and the difficulty that many cases present in terms of classification. For example, per some reports, behavioural disturbances are described to be ‘almost universally present’ in SD-Right patients,^23^^(see p. 2834)^ complicating their clinical differentiation from bvFTD. On this front, recent progress has been made in the form of clinical diagnostic criteria for SD-Right (referred to as semantic behavioural variant FTD^72^ or right-temporal variant FTD^23^) that emphasize early disturbances in empathy and social-emotional processing, marked semantic and behavioural changes, and relative sparing of motor-speech and visuospatial functions.^72^ The pattern of findings from our study largely align with these criteria, with the exception of visuospatial performance, where we found both bvFTD and SD-Right to display comparable difficulties. More importantly, although we have shown considerable overlap between bvFTD and SD across a range of cognitive and behavioural features, we do not by any means endorse that all such cases should be lumped together as simply FTD. Rather, we envisage that new methods to precisely identify pathological subgroups *in vivo* will enable us to better understand the pathophysiology of these cases and ultimately move towards curative drug therapies targeted to such pathologies. Nevertheless, there are strong grounds for considering SD as a unique clinico-pathological syndrome within the FTD spectrum. Such cases typically progress slowly, are rarely genetically determined^10^ and are strongly associated with a singular pathology [frontotemporal lobar degeneration-TAR DNA-binding protein (FTLD-TDP) Type C pathology] which is seldom found in other cases of FTD.^73^ It should be acknowledged, however, that clinico-pathological studies have, to date, not revealed any key features predictive of pathology in sporadic FTD cases beyond broad associations between SD with FTLD-TDP Type C pathology.^1^ The degree of association between SD and this specific pathology has further been shown to vary, perhaps reflecting some of the inherent overlap in clinical features with bvFTD,^74^ as highlighted by the present study. Syndromic classification in FTD is also challenging as patients present at different clinical stages depending on disease staging and progression.^13^ In this regard, the emergence of atypical symptoms with disease progression suggests the convergence of phenotypes with time.^13^ In the absence of longitudinal data, the current study is not positioned to address questions regarding phenotypic similarities and differences between FTD variants over time. However, we believe that transdiagnostic approaches can be leveraged by longitudinal studies to better characterize devolution of cognitive performance within the FTD spectrum, rather than viewing overlapping profiles as signalling conflicting diagnoses. Moreover, transdiagnostic approaches could prove critical in identifying predictive clinical features that definitively map onto pathology post-mortem. Longitudinal studies of this kind are extremely challenging and time-consuming to conduct but are of the utmost importance to accelerate our understanding of the links between phenotype, clinical features and underlying pathological drivers in bvFTD and SD.

In this vein, some limitations of our study warrant discussion. First, the majority of our patients have not yet come to autopsy nor had genetic information available, preventing us from conducting clinico-pathological and genetic explorations. Second, while we included multidomain cognitive testing, the detailed assessment of social-semantics, face-processing and social cognitive domains (e.g. theory of mind, empathy) was not available for these patients. In particular, measures of empathy and social cognition appear to aid in the early detection and differentiation of SD-Right from other FTD subtypes;^72^ therefore, future studies including detailed testing of these measures will be important to create robust data-driven solutions to address the independence and inter-dependence between socio-emotional and other cognitive/behavioural changes in FTD. Third, we constrained our imaging analyses to explore only grey matter changes and reported our VBM correlation results at an uncorrected threshold of *P* < 0.001. This threshold has been suggested as far more conservative than corrections for multiple comparisons and is increasingly adopted in studies of cognition and behaviour in neurodegenerative syndromes.^75,76^ Nevertheless, it is important that future studies use larger cohorts and integrate detailed clinical, cognitive–behavioural assessments with indices of pathological, genetic, natural history and multimodal neuroimaging (structural, functional and diffusion imaging) approaches. Finally, while our study showed that factor performance in FTD patients is not strongly associated with disease duration, it is important to conduct longitudinal explorations of cognitive heterogeneity to determine disease stages that critically predict the emergence of new symptoms. Together, these will allow to comprehensively model the multifaceted relationships between different mechanisms contributing to heterogeneity in FTD at different disease stages.^77^

Our findings hold a number of clinical implications. For practicing clinicians, detailed neuropsychological testing of FTD can improve characterization of the multidimensional FTD neurocognitive space, in turn, aiding the identification of measures that form highly reliable proxies of each dimension. By retaining measures that load highly on each dimension, one could arrive at a data-driven ‘reduced’ neuropsychological battery that retains important explanatory power to capture the key axes of cognitive–behavioural variation in neurodegenerative syndromes. Such a method has recently been implemented in post-stroke aphasia^78^ and would form an important avenue of exploration in FTD where age of disease onset, progression and aetiology patterns are highly variable. An improved characterization of the space may potentially translate to updated knowledge and training material, especially for clinicians outside of the clinical neurological setup. Mapping cognitive–behavioural variations to a unified FTD space affords potential opportunities to explore clinical management and symptomatic treatment options benefitting multiple disease groups rather than just one diagnostic category. Uncovering common dimensions of phenotypic change opens further possibilities to design treatment plans that harness common moderators of phenotypes to slow disease progression. By revealing the neural architecture of heterogeneous symptoms, we can consider the possibility of medical and functional restoration programmes targeting specific brain structural and functional networks. Our transdiagnostic approach also offers a fine-grained accompaniment to current categorical diagnostic approaches to capture symptomatic heterogeneity at individual, group and neural levels. Crucially, our findings can inform future revisions of the international diagnostic criteria for bvFTD and SD to accommodate graded differences in phenotypic presentation as characteristic of both syndromes. Adopting this approach in pathologically confirmed FTD patients can further refine our understanding of the primary syndrome and likely pathologies associated with mixed cognitive presentations of these syndromes. By creating such multidimensional spaces, it may also be possible to simplify longitudinal mapping of disease evolution and to explore disease progression in prototypical cases who may progress to show ‘atypical’ symptoms.^35^ This, in turn, may improve the capacity to provide tailored management and care information for patients, their families and carers. These remain important avenues for future work.

## Supplementary Material

fcac344_Supplementary_Data

## Data Availability

The ethical requirement to ensure patient confidentiality precludes public archiving of our data. Researchers who would like to access the raw data should contact the corresponding author (M.I.) who will liaise with the ethics committee that approved the study, and accordingly, as much data that are required to reproduce the results will be released to the individual researcher.

## Abbreviations

ACE-R =: Addenbrooke’s Cognitive Examination-Revised
ATL =: anterior temporal lobes
bvFTD =: behavioural variant frontotemporal dementia
CBI-R =: Cambridge Behavioural Inventory—Revised
CDR-FTLD SoB =: Clinical Dementia Rating—Frontotemporal Lobar Degeneration Sum of Boxes
FAST =: Facial Affect Selection Task
FTD =: frontotemporal dementia
FTLD-TDP =: frontotemporal lobar degeneration-TAR DNA-binding protein
FSL =: FMRIB Software Library
FWE =: Family-Wise Error
MNI =: Montreal Neurological Institute
NPI =: Neuropsychiatric Inventory
OFC =: orbitofrontal cortex
PCA =: principal component analysis
ROCF =: Rey–Osterrieth Complex Figure
ROI =: region of interest
SD =: semantic dementia
SYDBAT =: Sydney Language Battery
TMT B–A =: Trail Making Test parts B–A
VBM =: voxel-based morphometry

## References

[1] Bang J , Spina S, Miller BL. Frontotemporal dementia. Lancet. 2015;386(10004):1672–1682. 26595641 10.1016/S0140-6736(15)00461-4 PMC5970949

[2] Piguet O , Hornberger M, Mioshi E, Hodges JR. Behavioural-variant frontotemporal dementia: Diagnosis, clinical staging, and management. Lancet Neurol. 2011;10(2):162–172. 21147039 10.1016/S1474-4422(10)70299-4

[3] Ramanan S , Bertoux M, Flanagan E, et al Longitudinal executive function and episodic memory profiles in behavioral-variant frontotemporal dementia and Alzheimer's disease. J Int Neuropsychol Soc. 2017;23(1):34–43. 27751195 10.1017/S1355617716000837

[4] Chen Y , Huang L, Chen K, et al White matter basis for the hub-and-spoke semantic representation: Evidence from semantic dementia. Brain. 2020;143(4):1206–1219. 32155237 10.1093/brain/awaa057 PMC7191302

[5] Hodges JR , Patterson K. Semantic dementia: A unique clinicopathological syndrome. Lancet Neurol. 2007;6(11):1004–1014. 17945154 10.1016/S1474-4422(07)70266-1

[6] Lambon Ralph MA , Jefferies E, Patterson K, Rogers TT. The neural and computational bases of semantic cognition. Nat Rev Neurosci. 2017;18(1):42–55. 27881854 10.1038/nrn.2016.150

[7] Irish M , Kumfor F, Hodges JR, Piguet O. A tale of two hemispheres: Contrasting socioemotional dysfunction in right- versus left-lateralised semantic dementia. Dement Neuropsychol. 2013;7(1):88–95. 29213824 10.1590/S1980-57642013DN70100014 PMC5619550

[8] Kamminga J , Kumfor F, Burrell JR, Piguet O, Hodges JR, Irish M. Differentiating between right-lateralised semantic dementia and behavioural-variant frontotemporal dementia: An examination of clinical characteristics and emotion processing. J Neurol Neurosurg Psychiatry. 2015;86(10):1082–1088. 25511791 10.1136/jnnp-2014-309120

[9] Ding J , Chen K, Liu H, et al A unified neurocognitive model of semantics language social behaviour and face recognition in semantic dementia. Nat Commun. 2020;11(1):2595. 32444620 10.1038/s41467-020-16089-9 PMC7244491

[10] Seelaar H , Rohrer JD, Pijnenburg YA, Fox NC, van Swieten JC. Clinical, genetic and pathological heterogeneity of frontotemporal dementia: A review. J Neurol Neurosurg Psychiatry. 2011;82(5):476–486. 20971753 10.1136/jnnp.2010.212225

[11] Ranasinghe KG , Rankin KP, Lobach IV, et al Cognition and neuropsychiatry in behavioral variant frontotemporal dementia by disease stage. Neurology. 2016;86(7):600–610. 26802093 10.1212/WNL.0000000000002373 PMC4762418

[12] Vonk JMJ , Borghesani V, Battistella G, et al Verbal semantics and the left dorsolateral anterior temporal lobe: A longitudinal case of bilateral temporal degeneration. Aphasiology. 2020;34(7):865–885. 33012947 10.1080/02687038.2019.1659935 PMC7529336

[13] Murley AG , Coyle-Gilchrist I, Rouse MA, et al Redefining the multidimensional clinical phenotypes of frontotemporal lobar degeneration syndromes. Brain. 2020;143(5):1555–1571. 32438414 10.1093/brain/awaa097 PMC7241953

[14] Hardy CJ , Buckley AH, Downey LE, et al The language profile of behavioral variant frontotemporal dementia. J Alzheimers Dis. 2016;50(2):359–371. 26682693 10.3233/JAD-150806 PMC4740928

[15] Snowden JS , Harris JM, Saxon JA, et al Naming and conceptual understanding in frontotemporal dementia. Cortex. 2019;120:22–35. 31220614 10.1016/j.cortex.2019.04.027 PMC6838679

[16] Geraudie A , Battista P, Garcia AM, et al Speech and language impairments in behavioral variant frontotemporal dementia: A systematic review. Neurosci Biobehav Rev. 2021;131:1076–1095. 34673112 10.1016/j.neubiorev.2021.10.015 PMC12169595

[17] Harris JM , Jones M, Gall C, et al Co-occurrence of language and behavioural change in frontotemporal lobar degeneration. Dement Geriatr Cogn Dis Extra. 2016;6(2):205–213. 27350781 10.1159/000444848 PMC4913762

[18] Pozueta A , Lage C, Garcia-Martinez M, et al Cognitive and behavioral profiles of left and right semantic dementia: Differential diagnosis with behavioral variant frontotemporal dementia and Alzheimer's disease. J Alzheimers Dis. 2019;72(4):1129–1144. 31683488 10.3233/JAD-190877

[19] Strikwerda-Brown C , Ramanan S, Irish M. Neurocognitive mechanisms of theory of mind impairment in neurodegeneration: A transdiagnostic approach. Neuropsychiatr Dis Treat. 2019;15:557–573. 30863078 10.2147/NDT.S158996 PMC6388953

[20] Irish M , Hodges JR, Piguet O. Right anterior temporal lobe dysfunction underlies theory of mind impairments in semantic dementia. Brain. 2014; 137(Pt 4):1241–1253. 24523434 10.1093/brain/awu003

[21] Thompson SA , Patterson K, Hodges JR. Left/right asymmetry of atrophy in semantic dementia: Behavioral-cognitive implications. Neurology. 2003;61(9):1196–1203. 14610120 10.1212/01.wnl.0000091868.28557.b8

[22] Coyle-Gilchrist IT , Dick KM, Patterson K, et al Prevalence, characteristics, and survival of frontotemporal lobar degeneration syndromes. Neurology. 2016;86(18):1736–1743. 27037234 10.1212/WNL.0000000000002638 PMC4854589

[23] Erkoyun HU , Groot C, Heilbron R, et al A clinical-radiological framework of the right temporal variant of frontotemporal dementia. Brain. 2020;143(9):2831–2843. 32830218 10.1093/brain/awaa225 PMC9172625

[24] Josephs KA , Whitwell JL, Knopman DS, et al Two distinct subtypes of right temporal variant frontotemporal dementia. Neurology. 2009;73(18):1443–1450. 19884571 10.1212/WNL.0b013e3181bf9945 PMC2779005

[25] Chan D , Anderson V, Pijnenburg Y, et al The clinical profile of right temporal lobe atrophy. Brain. 2009;132(Pt 5):1287–1298. 19297506 10.1093/brain/awp037

[26] Husain M . Transdiagnostic neurology: Neuropsychiatric symptoms in neurodegenerative diseases. Brain. 2017;140(6):1535–1536. 28549134 10.1093/brain/awx115

[27] Dalgleish T , Black M, Johnston D, Bevan A. Transdiagnostic approaches to mental health problems: Current status and future directions. J Consult Clin Psychol. 2020;88(3):179–195. 32068421 10.1037/ccp0000482 PMC7027356

[28] Halai AD , Woollams AM, Lambon Ralph MA. Using principal component analysis to capture individual differences within a unified neuropsychological model of chronic post-stroke aphasia: Revealing the unique neural correlates of speech fluency, phonology and semantics. Cortex. 2017;86:275–289. 27216359 10.1016/j.cortex.2016.04.016 PMC5264368

[29] Butler RA , Lambon Ralph MA, Woollams AM. Capturing multidimensionality in stroke aphasia: Mapping principal behavioural components to neural structures. Brain. 2014;137(Pt 12):3248–3266. 25348632 10.1093/brain/awu286 PMC4240295

[30] Kozak MJ , Cuthbert BN. The NIMH research domain criteria initiative: Background, issues, and pragmatics. Psychophysiology. 2016;53(3):286–297. 26877115 10.1111/psyp.12518

[31] Ingram RU , Halai AD, Pobric G, Sajjadi S, Patterson K, Lambon Ralph MA. Graded, multidimensional intra- and intergroup variations in primary progressive aphasia and post-stroke aphasia. Brain. 2020;143(10): 3121–3135. 32940648 10.1093/brain/awaa245 PMC7586084

[32] Ramanan S , Roquet D, Goldberg ZL, et al Establishing two principal dimensions of cognitive variation in logopenic progressive aphasia. Brain Commun. 2020;2(2):fcaa125. 10.1093/braincomms/fcaa125 PMC7750924 33376980

[33] Lambon Ralph MA , Patterson K, Graham N, Dawson K, Hodges JR. Homogeneity and heterogeneity in mild cognitive impairment and Alzheimer's disease: A cross-sectional and longitudinal study of 55 cases. Brain. 2003;126(Pt 11):2350–2362. 12876147 10.1093/brain/awg236

[34] Fan JM , Gorno-Tempini ML, Dronkers NF, Miller BL, Berger MS, Chang EF. Data-Driven, visual framework for the characterization of aphasias across stroke, post-resective, and neurodegenerative disorders over time. Front Neurol. 2020;11:616764. 33447252 10.3389/fneur.2020.616764 PMC7801263

[35] Ramanan S , Irish M, Patterson K, Rowe JB, Gorno-Tempini ML, Lambon Ralph MA. Understanding the multidimensional cognitive deficits of logopenic variant primary progressive aphasia. Brain. 2022;145(9):2955–2966. 35857482 10.1093/brain/awac208 PMC9473356

[36] Jones D , Lowe V, Graff-Radford J, et al A computational model of neurodegeneration in Alzheimer's disease. Nat Commun. 2022;13(1):1643. 35347127 10.1038/s41467-022-29047-4 PMC8960876

[37] Cornblath EJ , Robinson JL, Irwin DJ, et al Defining and predicting transdiagnostic categories of neurodegenerative disease. Nat Biomed Eng. 2020;4(8):787–800. 32747831 10.1038/s41551-020-0593-y PMC7946378

[38] Rascovsky K , Hodges JR, Knopman D, et al Sensitivity of revised diagnostic criteria for the behavioural variant of frontotemporal dementia. Brain. 2011;134(Pt 9):2456–2477. 21810890 10.1093/brain/awr179 PMC3170532

[39] Neary D , Snowden JS, Gustafson L, et al Frontotemporal lobar degeneration: A consensus on clinical diagnostic criteria. Neurology. 1998;51(6):1546–1554. 9855500 10.1212/wnl.51.6.1546

[40] Knopman DS , Kramer JH, Boeve BF, et al Development of methodology for conducting clinical trials in frontotemporal lobar degeneration. Brain. 2008;131(Pt 11):2957–2968. 18829698 10.1093/brain/awn234 PMC2725027

[41] Wear HJ , Wedderburn CJ, Mioshi E, et al The Cambridge Behavioural Inventory revised. Dement Neuropsychol. 2008;2(2):102–107. 29213551 10.1590/S1980-57642009DN20200005 PMC5619578

[42] Cummings JL . The neuropsychiatric inventory: Assessing psychopathology in dementia patients. Neurology. 1997;48(5 Suppl 6):S10–S16. 10.1212/wnl.48.5_suppl_6.10s 9153155

[43] Mioshi E , Dawson K, Mitchell J, Arnold R, Hodges JR. The Addenbrooke's Cognitive Examination Revised (ACE-R): A brief cognitive test battery for dementia screening. Int J Geriatr Psychiatry. 2006;21(11):1078–1085. 16977673 10.1002/gps.1610

[44] Zarit SH , Orr NK, Zarit JM. The hidden victims of Alzheimer's disease: Families under stress. New York University Press; 1985.

[45] RStudio Team. RStudio: integrated development for R. RStudio, PBC; 2020. http://www.rstudio.com/

[46] Jolliffe IT . Principal component analysis. 2nd edn. Springer-Verlag; 2002.

[47] Smith SM . Fast robust automated brain extraction. Hum Brain Mapp. 2002;17(3):143–155. 12391568 10.1002/hbm.10062 PMC6871816

[48] Zhang YY , Brady M, Smith S. Segmentation of brain MR images through a hidden Markov random field model and the expectation-maximization algorithm. IEEE Trans Med Imaging. 2001;20(1):45–57. 11293691 10.1109/42.906424

[49] Andersson JLR , Jenkinson M, Smith S. Non-linear optimisation. University of Oxford FMRIB Centre; 2007.

[50] Andersson JLR , Jenkinson M, Smith S. Non-linear registration, aka spatial normalisation. University of Oxford FMRIB Centre; 2007.

[51] Shaw SR , El-Omar H, Ramanan S, et al Anhedonia in semantic dementia—Exploring right hemispheric contributions to the loss of pleasure. Brain Sci. 2021;11(8):998. 34439617 10.3390/brainsci11080998 PMC8392684

[52] Hwang YT , Strikwerda-Brown C, El-Omar H, et al “More than words”—Longitudinal linguistic changes in the works of a writer diagnosed with semantic dementia. Neurocase. 2021;27(3):243–252. 34003713 10.1080/13554794.2021.1924208

[53] Verdi S , Marquand AF, Schott JM, Cole JH. Beyond the average patient: How neuroimaging models can address heterogeneity in dementia. Brain. 2021;144(10):2946–2953. 33892488 10.1093/brain/awab165 PMC8634113

[54] Borroni B , Grassi M, Premi E, et al Neuroanatomical correlates of behavioural phenotypes in behavioural variant of frontotemporal dementia. Behav Brain Res. 2012;235(2):124–129. 22902293 10.1016/j.bbr.2012.08.003

[55] Williams GB , Nestor PJ, Hodges JR. Neural correlates of semantic and behavioural deficits in frontotemporal dementia. Neuroimage. 2005;24(4):1042–1051. 15670681 10.1016/j.neuroimage.2004.10.023

[56] Wong S , Balleine BW, Kumfor F. A new framework for conceptualizing symptoms in frontotemporal dementia: From animal models to the clinic. Brain. 2018;141(8): 2245–2254. 29762648 10.1093/brain/awy123

[57] Ducharme S , Price BH, Dickerson BC. Apathy: A neurocircuitry model based on frontotemporal dementia. J Neurol Neurosurg Psychiatry. 2018;89(4):389–396. 29066518 10.1136/jnnp-2017-316277 PMC6561783

[58] Seeley WW , Zhou J, Kim EJ. Frontotemporal dementia: What can the behavioral variant teach us about human brain organization? Neuroscientist. 2012;18(4):373–385. 21670424 10.1177/1073858411410354

[59] Tartaglia MC , Zhang Y, Racine C, et al Executive dysfunction in frontotemporal dementia is related to abnormalities in frontal white matter tracts. J Neurol. 2012;259(6):1071–1080. 22037958 10.1007/s00415-011-6300-x PMC3590313

[60] Shaw SR , El-Omar H, Roquet D, et al Uncovering the prevalence and neural substrates of anhedonia in frontotemporal dementia. Brain. 2021;144(5):1551–1564. 33843983 10.1093/brain/awab032

[61] Acosta-Cabronero J , Patterson K, Fryer TD, et al Atrophy, hypometabolism and white matter abnormalities in semantic dementia tell a coherent story. Brain. 2011;134(Pt 7):2025–2035. 21646331 10.1093/brain/awr119

[62] Ash S , Nevler N, Phillips J, et al A longitudinal study of speech production in primary progressive aphasia and behavioral variant frontotemporal dementia. Brain Lang. 2019;194:46–57. 31075725 10.1016/j.bandl.2019.04.006 PMC6656376

[63] Kravitz DJ , Saleem KS, Baker CI, Ungerleider LG, Mishkin M. The ventral visual pathway: An expanded neural framework for the processing of object quality. Trends Cogn Sci. 2013;17(1):26–49. 23265839 10.1016/j.tics.2012.10.011 PMC3532569

[64] Snowden JS , Harris JM, Thompson JC, et al Semantic dementia and the left and right temporal lobes. Cortex. 2018;107:188–203. 28947063 10.1016/j.cortex.2017.08.024

[65] Landin-Romero R , Kumfor F, Leyton CE, Irish M, Hodges JR, Piguet O. Disease-specific patterns of cortical and subcortical degeneration in a longitudinal study of Alzheimer's disease and behavioural-variant frontotemporal dementia. Neuroimage. 2017;151:72–80. 27012504 10.1016/j.neuroimage.2016.03.032

[66] Ossenkoppele R , Pijnenburg YA, Perry DC, et al The behavioural/dysexecutive variant of Alzheimer's disease: Clinical, neuroimaging and pathological features. Brain. 2015;138(Pt 9):2732–2749. 26141491 10.1093/brain/awv191 PMC4623840

[67] Townley RA , Graff-Radford J, Mantyh WG, et al Progressive dysexecutive syndrome due to Alzheimer's disease: A description of 55 cases and comparison to other phenotypes. Brain Commun. 2020;2(1):fcaa068. 10.1093/braincomms/fcaa068 PMC7325839 32671341

[68] Bocchetta M , Gordon E, Cardoso MJ, et al Thalamic atrophy in frontotemporal dementia—Not just a C9orf72 problem. Neuroimage Clin. 2018;18:675–681. 29876259 10.1016/j.nicl.2018.02.019 PMC5988457

[69] Halabi C , Halabi A, Dean DL, et al Patterns of striatal degeneration in frontotemporal dementia. Alzheimer Dis Assoc Disord. 2013;27(1):74–83. 22367382 10.1097/WAD.0b013e31824a7df4 PMC3389579

[70] Bocchetta M , Malpetti M, Todd EG, Rowe JB, Rohrer JD. Looking beneath the surface: The importance of subcortical structures in frontotemporal dementia. Brain Commun. 2021;3(3):fcab158. 10.1093/braincomms/fcab158 PMC8390477 34458729

[71] Gorno-Tempini ML , Hillis AE, Weintraub S, et al Classification of primary progressive aphasia and its variants. Neurology. 2011;76(11):1006–1014. 21325651 10.1212/WNL.0b013e31821103e6 PMC3059138

[72] Younes K , Borghesani V, Montembeault M, et al Right temporal lobe and socioemotional semantics: Semantic behavioural variant frontotemporal dementia. Brain. 2022;145(11): 4080–4096. 35731122 10.1093/brain/awac217 PMC10200288

[73] Hodges JR , Mitchell J, Dawson K, et al Semantic dementia: Demography, familial factors and survival in a consecutive series of 100 cases. Brain. 2010;133(Pt 1):300–306. 19805492 10.1093/brain/awp248

[74] Chare L , Hodges JR, Leyton CE, et al New criteria for frontotemporal dementia syndromes: Clinical and pathological diagnostic implications. J Neurol Neurosurg Psychiatry. 2014;85(8):865–870. 24421286 10.1136/jnnp-2013-306948

[75] Whitwell JL , Jack CR Jr, Boeve BF, et al Imaging correlates of pathology in corticobasal syndrome. Neurology. 2010;75(21):1879–1887. 21098403 10.1212/WNL.0b013e3181feb2e8 PMC2995388

[76] Sheelakumari R , Bineesh C, Varghese T, Kesavadas C, Verghese J, Mathuranath PS. Neuroanatomical correlates of apathy and disinhibition in behavioural variant frontotemporal dementia. Brain Imaging Behav. 2019;14(5):2004–2011. 10.1007/s11682-019-00150-3 PMC6942247 31273672

[77] Ahmed RM , Devenney EM, Irish M, et al Neuronal network disintegration: Common pathways linking neurodegenerative diseases. J Neurol Neurosurg Psychiatry. 2016;87(11):1234–1241. 27172939 10.1136/jnnp-2014-308350 PMC5099318

[78] Halai AD , Perez BDD, Stefaniak JD, Lambon Ralph MA. Efficient and effective assessment of deficits and their neural bases in stroke aphasia. Cortex. 2022;155:333–346. 36087431 10.1016/j.cortex.2022.07.014 PMC9548407

